# Intravesical oxybutynin for bladder capacity in children with spina bifida: the ‘Place de l’OXybutynine Intravésicale chez le Patient Enfant Neurologique’ (POXIPEN) trial protocol

**DOI:** 10.1111/bju.70058

**Published:** 2025-11-03

**Authors:** Marie Buzzi, Jonathan Epstein, Zahi Hatem, Nadine Juge, Charles Mazeaud, Jean‐Louis Lemelle, Nicolas Berte

**Affiliations:** ^1^ CHRU‐Nancy, INSERM, CIC, Epidémiologie Clinique Université de Lorraine Nancy France; ^2^ Inserm, INSPIIRE Université de Lorraine Nancy France; ^3^ CHRU‐Nancy, Pediatric Surgery Department Université de Lorraine Nancy France; ^4^ CHRU‐Nancy, Urology Department Université de Lorraine Nancy France

**Keywords:** urology, neurogenic bladder, spina bifida, paediatric surgery, randomised controlled trial

## Abstract

**Background:**

Neurogenic bladder is defined as a dysfunction of the bladder resulting from damage to the central or peripheral nervous systems. Its treatment is based on a progressive therapeutic escalation, rapidly involving invasive therapeutic procedures such as repeated intradetrusor injections and surgery. Given the risk of repeated general anaesthesia in children, there is a need for non‐invasive treatment for young patients who do not respond to or have adverse effects from oral anticholinergic treatment. The ‘Place de l’OXybutynine Intravésicale chez le Patient Enfant Neurologique’ (POXIPEN) trial aims to assess the efficacy of intra‐vesical oxybutynin on bladder capacity in a sample of French children with neurogenic bladder.

**Study Design:**

The POXIPEN is a multicentre, randomised, double‐blind, placebo‐controlled trial.

**Endpoints:**

The primary outcome is change in maximal bladder capacity after treatment. Secondary outcomes include changes in voiding, urodynamic and ultrasound parameters. We will also assess changes on quality of life and usability of the product.

**Patients and Methods:**

We aim to randomly assign 60 children with neurogenic bladder secondary to spina bifida and deemed non‐responders to first‐line treatment with oral anticholinergics, to receive intravesical oxybutynin (IVO) or placebo for 4 weeks. Recruitment will start in September 2025. It will be the first prospective study to evaluate the efficacy of IVO in children, with a high level of evidence provided by its design. If IVO proves effective, it could lengthen the delay in therapeutic escalation to invasive procedures, thereby reducing the risk of complications associated with general anaesthesia in children with neurogenic bladder.

**Trial registration:**

This trial is registered with the European Union (EU) Clinical Trials Information System (CTIS) under EU CT Number: 2022–501 902–36‐00 (approved 09.01.2025) and ClinicalTrials.gov under identifier NCT07027020 (registered 18.06.2025).

**Abbreviations**
(S)AE: (Serious) Adverse Event
CAP: Centre Anti‐Poison
CHRU: Centre Hospitalier Régional Universitaire
eCRF: electronic Case Report Form
ICIQ‐UI‐SF: International Consultation on Incontinence Questionnaire‐Urinary Incontinence Short Form
IVO: IntraVesical Oxybutynin
KIDSCREEN‐10: 10‐item KIDSCREEN questionnaire
POXIPEN: Place de l’OXybutynine Intravésicale chez le Patient Enfant Neurologique (Role of Intravesical OXybutynin in Neurological Paediatric Patients)
RCT: Randomised Controlled Trial
UMUX: Usability Metric for User eXperience

## Background

Lumbosacral pathologies, such as spina bifida, can affect bladder innervation and lead to a neurogenic bladder, often characterised by a non‐compliant, hyperactive, and small capacity bladder [1]. These dysfunctions usually lead to incontinence and obstruction of urine evacuation, eventually resulting in renal failure. The current therapeutic approach aims to improve urine evacuation and reduce intravesical pressure and progressively involves: (i) intermittent urinary catheterisation, (ii) oral anticholinergics, (iii) intradetrusor injections of onabotulinumtoxinA (Botox®, AbbVie Inc., North Chicago, IL, USA) and (iv) surgery.

As an anticholinergic, oxybutynin is usually prescribed orally. However, there are some contraindications and side effects associated with its oral administration, including important frequent neurological effects such as cognitive impairment [2], as well as a risk of drug resistance, requiring therapeutic escalation. The next step, intradetrusor injections of onabotulinumtoxinA, is invasive and must be performed under general anaesthesia in children. As for surgical procedures, although effective, they are irreversible and responsible for morbidity and mortality.

Since 1989 and the first study by Brendler et al. [3], the efficacy of intravesical oxybutynin (IVO) on detrusor overactivity and bladder hypocompliance has been reported in several studies [4, 5, 6, 7, 8, 9]. In 2008, a literature review conducted by Guerra et al. [10] focusing on children with a neurological condition who were resistant or had significant side effects with oral oxybutynin, showed improved bladder compliance and reduced bladder pressure with IVO. However, their level of evidence was not sufficient to support a change in practices. The 2016 study by Schröder et al. [11] was the first multicentre randomised controlled trial (RCT) comparing IVO with oral oxybutynin in adults. It showed excellent results, both in terms of efficacy in increasing bladder capacity, and security, with fewer side effects in the IVO group.

Although IVO appears to be a promising alternative to oral treatment in both adults and children, it is not yet included in international recommendations for the treatment of neurogenic bladder. The issue of therapeutic escalation, which leads affected patients, sometimes very young, to undergo multiple surgeries, is an important one, as the repeated use of general anaesthesia in children has been shown to affect their mental development [12, 13].

Actual recommendations on the management of neurogenic bladder in children – including guidelines from the European Association of Urology [14] – include the administration of anticholinergics as soon as possible, including in newborns [14, 15, 16]. However, in France, oxybutynin is, to date, only available in tablet form, which very young children may struggle to ingest. Another anticholinergic, solifenacin, is available in liquid form. Nevertheless, French authorities have considered the evidence insufficient to support its reimbursement for paediatric neurogenic bladder, unlike other countries in which solifenacin has been approved for paediatric use. Besides, and like other oral anticholinergics, solifenacin is still associated with systemic side effects, partly due to greater variability in serum concentrations [17, 18]. Other therapeutic alternatives, such as beta‐3 agonists (e.g., mirabegron) are also only available in tablet form and data on their efficacy in children remain limited. IVO would thus enable early anticholinergic treatment to be easily administered in newborns and young children undergoing intermittent catheterisation. In Germany, where IVO is already widely used in very young children who have failed oral anticholinergic therapy, its effectiveness seems highly promising and well accepted by patients and their families. However, it is not yet prescribed as an alternative to oral oxybutynin in other countries, such as France, in which it has only been granted a temporary authorisation for nominative use in March 2021.

As a consequence, we hypothesise that IVO may be a viable treatment option for the treatment of neurogenic bladder in paediatric patients who have failed anticholinergic therapy prior to a therapeutic escalation requiring an invasive procedure. We designed the ‘Place de l’OXybutynine Intravésicale chez le Patient Enfant Neurologique’ (POXIPEN; ‘Role of Intravesical OXybutynin in Neurological Paediatric Patients’) study, the first RCT to assess the efficacy of IVO on bladder capacity in a sample of French children with neurogenic bladder.

## Methods

### Study Design

The POXIPEN trial is a national multicentre randomised double‐blind placebo‐controlled trial, involving 19 French paediatric urological surgery departments. The list of the centres involved is available on Clinicaltrial.gov, where the trial is registered as NCT07027020.

### Objectives

The main objective of the POXIPEN trial is to assess the efficacy of IVO vs placebo on maximal bladder capacity in children with neurogenic bladder secondary to myelomeningocele (spina bifida), performing intermittent catheterisation, and for whom oral anticholinergic therapy has been deemed ineffective or associated with adverse effects.

Secondary objectives are:

- To assess evolution of maximum bladder pressure after IVO (or placebo);
- To assess incontinence (i.e., time to clinical failure of treatment as perceived by the patient) after IVO (or placebo);
- To describe treatment tolerance and adverse effects;
- To assess the number of responders and continent patients after IVO (or placebo);
- To assess usability of IVO;
- To assess changes in patients’ quality of life after IVO (or placebo);
- To describe changes in patients’ voiding diary data after IVO (or placebo);
- To describe changes in urodynamic and in renal ultrasound parameters after IVO (or placebo);
- To identify patients’ characteristics associated with a greater likelihood of responding to IVO.

### Population and Sampling

The POXIPEN trial aims to include paediatric patients aged ≥6 years with neurological detrusor hyperactivity secondary to myelomeningocele (spina bifida) for whom oral anticholinergic therapy has been deemed ineffective or associated with adverse effects.

Inclusion criteria are as follows:

- Age between 6 and 17 years;
- Detrusor hyperactivity secondary to spina bifida, confirmed by a urodynamic test carried out within the last 6 months, according to the International Children's Continence Society (ICCS) criteria [19];
- Use of intermittent catheterisation for at least 6 weeks and at least three times a day;
- Ability and willingness to perform catheterisation and intravesical instillation (patient or parents);
- Failed treatment with one or more anticholinergic agents, defined by a response deemed insufficient by the investigator after at least 4 weeks of treatment at optimal dose, inability to take oral oxybutynin, or adverse effects deemed intolerable by the patient and/or the investigator. Reason for deemed inefficacy or intolerance will be systematically recorded at the inclusion visit;
- Renal ultrasound carried out in the last 2 months;
- Cystomanometry (with maximum volume and maximum pressure) carried out in the last 6 months.

Exclusion criteria are:

- Allergy to any component of the product under evaluation;
- Contraindication to oxybutynin (hypersensitivity, myasthenia, glaucoma, functional or organic gastrointestinal obstruction, severe gastrointestinal disease, subvesical obstruction, ongoing treatment with anticholinergic drugs for another indication that cannot be discontinued, polyuria of non‐vesical origin, concomitant oxygen therapy);
- Hyperthyroidism, coronary artery disease, congestive heart failure, cardiac arrhythmia, tachycardia, uncontrolled high blood pressure;
- Ongoing treatment with bisphosphonates, P450 cytochromes or cholinesterase inhibitors;
- Women of childbearing age without highly effective contraception, pregnant woman or nursing mother;
- Intradetrusor injection of onabotulinumtoxinA in the last 6 months.

All eligible patients will be screened for inclusion and exclusion criteria during a routine follow‐up visit to one of the participating centres. If a patient is being treated with oral anticholinergics, it will be stopped at the time of inclusion, to ensure a 7‐day wash‐out period before treatment initiation.

Randomisation will be performed by a centralised computer program, stratified by age group (6–11 years, ≥12 years) with the use of a permuted block design of size 10. Patients will be randomised to IVO vs placebo with a 1:1 allocation.

### Intervention

The experimental group will receive oxybutynin hydrochloride (VESOXX®, FARCO‐PHARMA GmbH, Cologne, Germany) at a concentration of 1 mg/mL in 10 mL syringes ready for intravesical instillation for 4 weeks. The treatment will be instilled at the end of evacuation catheterisation, and the solution will be retained in the bladder until the next scheduled catheterisation, with a minimum dwell time of 30 min to ensure sufficient mucosal exposure. The total daily dose will be 0.4 mg/kg/day. If the calculated per‐instillation dose does not exceed 10 mg, the daily dose will be divided into two instillations. If it exceeds 10 mg per instillation, the daily dose will be divided into three equal instillations, to remain within the per‐dose cap. The maximum daily dose will be 20 mg/day for patients aged <12 years, and 30 mg/day for patients aged ≥12 years. The first administration will be carried out by a hospital nurse in a double‐blind setting during a therapeutic education session. Subsequent instillations will be carried out by the patient or his/her parents. To ensure consistency, instillations will be spaced at approximately 12‐h intervals (±2 h) when two instillations are required, and at approximately 8‐h intervals (±3 h) when three instillations are required. This compromise allows for standardisation of dosing intervals while accommodating family and school logistics. The usual catheterisation schedule will not be modified, in order to avoid altering bladder behaviour and to enhance acceptability for families.

The control group will receive the same volume of 0.9% sodium chloride in the same modalities as the intervention group. As the modalities of administration and packaging will be the same in the two arms, neither the patient, the investigator nor the monitor will be informed of the treatment assigned.

### Endpoints

The primary outcome is change in maximum bladder capacity after 4 weeks of treatment (end of follow‐up). This parameter is considered by experts to be an objective marker of improvement in neurogenic bladder. It will be measured by cystomanometry and is defined by the maximum bladder volume (mL) recorded after filling via the urethral catheter, which may occur under one of the following conditions: (i) when the patient is given permission to urinate, (ii) when detrusor pressure reaches 40 cmH₂O, (iii) when the expected maximal bladder volume for age is reached, or (iv) when the patient reports an irrepressible urge to urinate. In younger patients, urination occurs spontaneously, and this value will be obtained *a posteriori* by curve analysis. Change will be assessed between two measurement times: one before initiation of treatment, performed <6 months prior (i.e., reference cystomanometry), at best in the absence of oral anticholinergic treatment, and the other at the end of treatment, under the same conditions. Cystomanometers will be calibrated at the start of each procedure.

Secondary outcomes will be:

- The 4‐week change in maximum bladder pressure, measured by cystomanometry.
- Time to treatment failure within 28 days, defined on the basis of criteria available in the literature (at least one of three criteria):
  - Treatment deemed ineffective by the patient or practitioner;
  - A <50% reduction of incontinence episodes reported in the voiding diary;
  - Intolerable side effects reported by the patient.
- Occurrence of adverse events (AEs; including digestive, psychiatric, neurological, skin, urological and pain‐related disorders) and their time of onset, as recorded by the patient in his/her treatment journal (Data S1) or by the practitioner during follow‐up visits.
- Proportion of responders, i.e., patients who had a ≥50% reduction in urinary incontinence episodes recorded in their voiding diary [20], after 4 weeks of treatment, with no intolerable AEs.
- Proportion of continent patients, i.e., patients who had a 100% reduction in urinary incontinence episodes, after 4 weeks of treatment [20].
- Standardised difference in patient's quality of life, as assessed by changes in the International Consultation on Incontinence Questionnaire‐Urinary Incontinence Short Form (ICIQ‐UI‐SF) [21] and the 10‐item KIDSCREEN questionnaire (KIDSCREEN‐10) [22] scores between the inclusion visit and the end of follow‐up.
- Changes in voiding diary parameters, as assessed by the change in the number and volume of urinary catheterisations over 72 h during the week preceding each follow‐up visit. If treatment is effective, voided volume should increase.
- Changes in urodynamic parameters between the reference cystomanometry and the cystomanometry performed at the end of treatment:
  - Bladder compliance (mL/cmH_2_O),
  - Minimum filling volume causing uninhibited detrusor contraction,
  - Detrusor leak point pressure (cm H_2_O),
  - Volume of bladder filling during urine loss (mL).

To ensure consistency and standardisation across centres, all participating investigators will be asked to perform both cystomanometries under the same conditions for each patient, regarding bladder filling rates and patient positioning.

- Changes in renal ultrasound parameters between the reference renal ultrasound (performed <2 months prior to treatment initiation), and the one performed at the end of treatment:
  - Pyelon anteroposterior diameter (mm),
  - Ureter diameter (mm).

If treatment is effective, a reduction in both diameters could be observed, as a result of the decrease of pressure in the renal system.

- Usability of the product, measured by the Usability Metric for User eXperience (UMUX)‐LITE scale [23] coupled with seven ad hoc questions specific to the treatment and disease under study, measured on a 7‐point Likert scale (from strongly disagree to strongly agree; Data S2).

### Data Collection and Patients’ Follow‐Up

Three follow‐up visits are planned for each patient. Visit 1 (i.e., Day 1 of treatment) will include a measurement of quality of life using the ICIQ‐UI‐SF and KIDSCREEN‐10 questionnaires, a clinical examination, and an evaluation of the voiding diary completed over a 72‐h period before the visit. The treatment kit assigned by the centralised randomisation system will be given to the patient to cover the 4 weeks of treatment, and the first administration will be performed by a nurse, after a therapeutic education session. Each patient will receive a treatment journal to record the information needed to monitor compliance (dates and times of administration, observations), as well as adverse effects. If the patient is a child, parents will be asked to complete the document.

Visit 2 will take place after 2 weeks of treatment. It will include a clinical examination and an evaluation of the voiding diary and treatment journal.

Visit 3 will take place at the end of the 4 weeks of treatment. It will include a clinical examination and an evaluation of the voiding diary and treatment journal. If the patient has already discontinued the treatment before the visit, the date of discontinuation will be noted. Otherwise, the treatment will be interrupted on this visit. A cystomanometry will be carried out under the same conditions as the one carried out before the inclusion visit, as well as a renal ultrasound. Quality of life and usability of the product will be assessed.

A urine pregnancy test will be performed at each visit in female patients of childbearing age.

Between visits, patients will be asked to record the number and volume of catheterisations, as well as urine leakage for 72 consecutive hours in their voiding diary provided during the inclusion visit (Data S3).

Treatment or placebo may be stopped prematurely at any time in case of serious adverse effect, if the investigator deems it necessary for the patient's safety, or if the patient wishes to discontinue treatment. In this case, only the next scheduled visit is maintained (i.e., Visit 2 or 3) and the procedures planned for Visit 3 will be carried out to collect outcomes. The patient will continue to receive usual medical care.

A follow‐up call will be scheduled 7 days after the last consultation, to assess any residual effect of the treatment or placebo.

### Data Management and Monitoring

During the trial, the investigator, or any person designated in writing in the task delegation form, will document an electronic case report form (eCRF) for each patient included in the study. The eCRF will be set up and managed by the Nancy Centre Hospitalier Régional Universitaire (CHRU). The investigator will be responsible for the quality, accuracy and relevance of the data collected in the eCRF.

Identification of included patients will be limited to an identification code assigned on inclusion composed as follows: Centre number – Inclusion number in chronological order.

Compliance with the clinical investigation plan and security regulations, as well as data accuracy, will be monitored by the Clinical Research Associate mandated by the sponsor (Nancy CHRU). The full database will be stored on the Nancy CHRU server for 15 years after the end of the trial. An audit could be conducted at any time by independent experts on the sponsor demand.

### Safety Profile

All patients included in the study will have already received oral anticholinergic treatment, which reduces the risk of allergy to oxybutynin. In addition, any contraindication to the use of oral anticholinergic treatment also contraindicates participation in the present study, which should reduce the risk of serious AEs (SAEs).

Intravesical administration is associated with better bioavailability of oxybutynin compared to oral administration, as there is no first intestinal passage. This results in a reduction of its main metabolite, N‐desethyloxybutynin, which is less effective and has significant side effects [24]. Previous studies showed that the occurrence of adverse effects was lower with intravesical administration, as compared with oral oxybutynin [11, 15, 25, 26, 27, 28]. In the study by Schröder et al. [11], the adverse effects specifically found in the IVO group were: pain at the instillation site, headaches, abdominal pain, urinary urgency, decreased sweating, and hypotension. The adverse effects found in both modes of administration of oxybutynin were: dry mouth, constipation, dizziness, fatigue, dry eyes, attention disorders, drowsiness, apathy, and delayed urination. Finally, the expected risks of intravesical instillation, both for the oxybutynin and placebo groups are as follows: pain at the instillation site, urinary urgency and UTI.

Adverse effects will be systematically documented at each visit using the treatment journal. In case of fever, the patient will be prescribed an emergency urine culture. If positive, the patient will be urgently referred to a doctor at his/her centre to assess the need for antibiotics.

In our study population, where the next therapeutic step is often intradetrusor injections of onabotulinumtoxinA (an invasive procedure requiring anaesthesia), we considered placebo as the most appropriate comparator to establish the true effect size of IVO beyond natural variability. The 4‐week treatment period was chosen as a compromise to provide sufficient time to assess efficacy while avoiding unnecessarily long exposure to an ineffective treatment. All participants will continue to receive standard conservative management during this period, including intermittent catheterisation and surveillance. Investigators may request unblinding at any time in the event of a severe medical emergency potentially related to the study drug. Isolated incontinence episodes will not in themselves be considered a medical emergency requiring unblinding, as they are expected in children who have previously failed or not tolerated oral anticholinergics.

The unblinding procedure will be managed by the Lyon Hospitals Poison Control Centre (Centre Anti‐Poison [CAP]), which will provide access to information on the treatment of participants in the event of a medical emergency. The investigator may request unblinding for any reason deemed essential to the therapeutic management of the patient, by contacting the CAP. Any emergency unblinding must be followed by a SAE report.

There is no evidence in the literature suggesting any additional risk beyond the study period. Repeated IVO should not result in an accumulation of oxybutynin in the body, as its elimination half‐life is short (2 h). However, patients and their parents will be asked to report any suspected delayed events to the investigator, and a follow‐up call will be scheduled 7 days after the last consultation, to ensure that there are no residual effects of the treatment. As patients with a neurogenic bladder are systematically monitored as part of their pathology follow‐up, any delayed adverse effects will be identified by the investigators.

An independent Data and Safety Monitoring Board (DSMB), composed of two paediatric surgeons and one biostatistician, will be responsible for overseeing the trial and addressing any safety concerns that may arise.

### Sample Size

Schröder et al. [11] found a 32% increase in maximum bladder capacity in adults using IVO compared with baseline values (*P* < 0.001). In our target population, Raes et al. [29] found a 33% increase in maximum bladder capacity when treated with tolterodine. As patients included in our study already use catheterisation, there is no reason to expect a strong placebo effect from instillation. Given the lack of precise data on the effect of IVO in our target population and based on experts’ opinion, we expect a 33% increase in maximum bladder capacity in the treated group, a 5% increase in the placebo group, a mean (SD) initial bladder capacity of 170 (80) mL, and a correlation between measurements for the same patient of 0.8. To obtain a power of 0.9 with a type I error risk set at 0.05, 26 patients per group are needed. To allow for a margin of error, we rounded up the total expected sample size to 60.

### Statistical Analyses

For the primary outcome, an intention‐to‐treat analysis will be conducted to assess the difference between the two groups in the evolution of maximum bladder capacity, using a linear mixed model accounting for repeated measurement, age stratification, and treatment exposure as a binary variable. A secondary exploratory analysis will be conducted to account for any imbalance that may occur between groups despite randomisation due to the small sample size. Multivariate linear regression will be performed, adjusted for age and gender.

Given the small sample size and the short duration of follow‐up for each patient, there should be little or no missing data. For the primary outcome, patients lost to follow‐up will be assigned the value measured during the last cystomanometry performed (i.e., last observation carried forward).

No intermediate analysis is planned given the small sample size.

Two secondary outcomes will be analysed following a fixed sequence in a priori ordered hypotheses:

- Maximum bladder pressure after 4 weeks of treatment: the two groups will be compared using the same methodology as for the primary outcome;
- Time to treatment failure: the two groups will be compared using a two‐tailed log‐rank test. If a patient is lost to follow‐up, the date of treatment failure will be the date of last news plus 1 day.

Occurrence of AEs will be compared in the two groups using a Fisher's test with a significance threshold = 0.05.

The proportions of responders and continent patients after 4 weeks of treatment will be calculated with their 95% CI.

Standardised differences in patients’ quality of life assessed with the KIDSCREEN‐10 and ICIQ‐UI‐SF questionnaires will be compared between the two groups using a Wilcoxon test, with a significance threshold = 0.05. As recommended by the KIDSCREEN‐10 developers, an additional subgroup analysis will be performed to distinguish children aged 6–7 years from those aged 8–17 years.

Changes in voiding diary elements, urodynamic parameters, and renal ultrasound parameters after 4 weeks of treatment will be compared using a Wilcoxon test, with a significance threshold = 0.05.

Product usability will be assessed using the linear transformation of the UMUX‐LITE score proposed by the authors and comparing the average scores in both groups to reference scores (e.g., a score >80 is associated with good usability). A median score will be calculated in the two groups for the supplementary usability questions specific to our study.

Finally, the association between responder status and the following variables will be tested using a Fisher's test, with a significance level = 0.05:

- Cause of discontinuation of oral anticholinergic treatment (inability to swallow, no effect on bladder hyperactivity, intolerable side effects);
- Neurological status (walking vs non‐walking, presence of a ventriculoperitoneal shunt valve);
- Concomitant diseases;
- Concomitant treatment.

All analyses will be conducted with R statistical software, version 4.0.3 (R Foundation for Statistical Computing, Vienna, Austria).

### Ethics

Eligible patients and their legal guardians will be informed about the trial objectives, the possibility to have access to and to modify their personal data, and to indicate that they do not want their personal data used for research purpose. This information will be summarised in a written document. In case both parents have parental authority, the inclusion of their child will only be possible if both written consents are obtained. If only one parent is still alive or has parental authority, or when the patient is represented by a legal guardian, only one written consent will be necessary and a copy of the court judgement will be requested for documentation in the patient's file. Minor patients will receive information in a way that is adapted to their ability to understand. They will be informed orally by the investigator and will receive an information letter adapted to their age. Their personal consent for their participation in the study will be sought, and their assent will be documented in the consent form. If the patient reaches the age of majority during his/her participation in the trial, a new information note will be given to him/her, and he/she will have to sign a new consent form at the next visit.

The Comité de protection des personnes (CPP), i.e., French equivalent for the Institutional Review Board, has reviewed the trial protocol under the number 2022‐501902‐36‐00 and approved it. The declaration of compliance with the reference methodology MR001 has been sent to Commission Nationale de l’Informatique et des Libertés (CNIL) on 21 July 2023. The Agence Nationale de Sécurité du Médicament et des produits de santé (ANSM), i.e., the French drug safety agency, has granted authorisation for the trial on 2 January 2025.

## Discussion

### Current Status of the Trial

The trial is funded by a grant from the French Ministry of Health (PHRC‐N‐2021), and FARCO PHARMA laboratories, who will also supply experimental treatment and placebo. The study also received financial support from the Association Nationale Spina Bifida Handicaps (ASBH) and institutional support from the Société Francophone d’Urologie Pédiatrique et de l’Adolescent (SFUPA).

The mandatory French ethic committees granted authorisation for the trial.

The eCRF is achieved. The trial recruitment will be launched in September 2025 and will end on September 2027.

The trial protocol was written according to the Standard Protocol Items: Recommendations for Interventional Trials (SPIRIT) guidelines (Data S5) [30].

### Implications

The POXIPEN trial will be the first RCT to evaluate the efficacy of IVO on neurogenic bladder in children. It should provide a high level of evidence of the benefit of IVO in this patient population. If IVO proves effective in improving maximal bladder volume and pressure, it could lengthen the delay in therapeutic escalation to intravesical onabotulinumtoxinA injection, thereby reducing the risk of complications associated with general anaesthesia in children. Besides, reducing the incidence of bladder leakage could improve patients’ quality of life. For ethical and regulatory reasons, our study population is limited to children aged ≥6 years. If efficacy and safety are demonstrated in this population, future studies should be designed to evaluate IVO in younger children, who represent an important target population. From an individual point of view, if IVO is effective for a patient during the study, it will be possible for the referring investigator to prescribe this treatment as part of a compassionate use after the study. The French regulatory framework could then evolve towards a marketing authorisation by health authorities, thus changing the way paediatric patients with neurogenic bladder disease are managed.

A long‐term evaluation of this treatment, including a medico‐economic evaluation, could be carried out in a second phase, following completion of this study.

## Ethics Statement

This study has been approved by French ethics authorities through the European Union (EU) Clinical Trials Information System (CTIS). The committee's reference number for this approval is EU CT Number: 2022‐501902‐36‐00. Written and informed consent will be obtained from participants’ legal guardians (Data S4) and non‐opposition of minor patients will be sought prior to any study‐related procedures.

## Consent for Publication

Not applicable.

## Disclosure of Interests

The authors declare that they have no competing interests.

## Funding

This study was supported by a grant from the French Ministry of Health (PHRC‐N‐2021), and FARCO PHARMA laboratories, who are making a financial contribution to the project, as well as supplying experimental treatment and placebo. The study also received financial support from the Association Nationale Spina Bifida Handicaps (ASBH) and institutional support from the Société Francophone d’Urologie Pédiatrique et de l’Adolescent (SFUPA). Funders were not involved in the conceptualisation, design, decision to publish, or preparation of the manuscript.

## Author Contributions

Nicolas Berte, Jean‐Louis Lemelle, Jonathan Epstein, Zahi Hatem and Charles Mazeaud have conducted conceptualisation and design of the protocol. Marie Buzzi wrote the first draft of the manuscript. All authors edited, read, and approved the final manuscript.

## Administrative Information

Protocol version: version 1.4 (24/12/18). The protocol may be modified during the trial. The new version will be submitted to the sponsor and may be subject to a request for advice or authorisation from the relevant regulatory authorities. Name and contact information for the trial sponsor: Centre Hospitalier Régional Universitaire de Nancy, Avenue du Maréchal de Lattre de Tassigny, 54 035 NANCY Cedex.

Role of sponsor: The sponsor was not involved in the conceptualisation, design, decision to publish, or preparation of the manuscript.

## Supporting information


**Data S1** Extract from the treatment journal.
**Data S2** Supplementary usability items.
**Data S3** Extract from the voiding diary.
**Data S4** Model consent form.
**Data S5** The Standard Protocol Items: Recommendations for Interventional Trials (SPIRIT) checklist.

## Data Availability

Data sharing is not applicable to this article as no datasets were generated or analysed during the current study.
